# Effects of flowing water stimulation on hormone regulation during the maturation process of *Conger myriaster* ovaries

**DOI:** 10.3389/fphys.2024.1404834

**Published:** 2024-05-02

**Authors:** Zhengcheng Li, Rucong Liu, Jingwei Liu, Zhixin Jiang, Xubing Ba, Kang Li, Liping Liu

**Affiliations:** ^1^ China-ASEAN Belt and Road Joint Laboratory on Mariculture Technology (Shanghai), Shanghai Ocean University, Shanghai, China; ^2^ Center for Ecological Aquaculture (CEA), Shanghai Ocean University, Shanghai, China; ^3^ Shanghai Engineering Research Center of Aquaculture, Shanghai Ocean University, Shanghai, China

**Keywords:** conger eel, artificial reproduction, swimming, ovarian development, hormone regulation

## Abstract

Conger eel (*Conger myriaster*) is an economically important species in China. Due to the complex life history of the conger eel, achieving artificial reproduction has remained elusive. This study aimed to explore the effect of water stimulation on hormonal regulation during the artificial reproduction of conger eel. The experiment was divided into four groups: A1 (no hormone injection, still water), A2 (no hormone injection, flowing water), B1 (hormone injection, still water), and B2 (hormone injection, flowing water). The flowing water group maintained a flow velocity of 0.4 ± 0.05 m/s for 12 h daily throughout the 60-day period. Steroid hormone levels in the serum and ovaries of conger eels were analyzed using UPLC-MS/MS and ELISA on the 30th and 60th days of the experiment. The relative expression levels of follicle-stimulating hormone (*FSHβ*) and luteinizing hormone (*LHβ*) in the pituitary were determined by quantitative PCR. The results showed a significantly lower gonadosomatic index (GSI) in B2 compared to B1 (*p < 0.05*) on the 30th day. FSH was found to act only in the early stages of ovarian development, with water stimulation significantly enhancing FSH synthesis (*p < 0.05*), while *FSHβ* gene was not expressed after hormone injection. Conversely, LH was highly expressed in late ovarian development, with flowing water stimulation significantly promoting LH synthesis (*p < 0.05*). Serum cortisol (COR) levels were significantly higher in the flowing water group than in the still water group (*p < 0.05*). Furthermore, estradiol (E2) content of B2 was significantly lower than that of B1 on the 30th and 60th day. Overall, flowing water stimulation enhanced the synthesis of FSH in early ovarian development and LH in late ovarian development, while reducing E2 accumulation in the ovaries. In this study, the effect of flowing water stimulation on hormone regulation during the artificial reproduction of conger eel was initially investigated to provide a theoretical basis for optimizing artificial reproduction techniques.

## 1 Introduction

Conger eel (*Conger myriaster*) is a highly economically valuable demersal fish found in the Pacific Northwest (Ma et al., 2018).They are often referred to as sand eels due to their predominant habitat in coastal mud, sand, and gravel substrates (Ma et al., 2018). Currently, artificial propagation of eels remains challenging, with eel culture industries relying solely on wild-caught larvae resources (Horie et al., 2010). Previous studies have successfully obtained and hatched fertilized eel eggs through exogenous hormone-induced maturation. However, challenges persist, including high larval mortality rates and poor quality of fertilized eggs (Utoh et al., 2010b; Horie et al., 2010). Therefore, optimizing the artificial breeding techniques is of great importance for both research and industrial practice in eels.

The conger eel is a deep-sea migratory fish (Hao et al., 2020). The ovaries of the conger eel gradually mature during migration and lay eggs near the spawning grounds (Okamura et al., 2000; Li et al., 2018; Xiaolong et al., 2021). Under captive breeding condition, the ovarian development of female broodstock stops at the secondary yolk globule stage (Utoh et al., 2010a; Chiba et al., 2010). The development of ovaries in eels only occurs when exogenous hormones are injected (Utoh et al., 2010a; Chiba et al., 2010). Swimming behavior is the main way to realize the physiological activity of migratory fish, which is important for individual survival and population reproduction (Liu et al., 2015). Swimming can promote gonadal maturation in male eels, and promote lipid accumulation in female eels' oocytes (Couillard et al., 1997; Palstra et al., 2007; Palstra et al., 2009; Palstra et al., 2010b). The development of the gonads in teleost is controlled by the hypothalamic-pituitary-gonadal (HPG) axis (Kr et al., 2020). Inhibition of gonadal development in eels is caused by dopamine suppression and gonadotropin-releasing hormone deficiency, which in turn inhibits the synthesis of follicle-stimulating hormone (FSH) and luteinizing hormone (LH) (Palstra et al., 2009; Wang et al., 2015). FSH primarily promotes the biosynthesis of testosterone T) and estradiol (E2), which are essential for spermatogenesis and oogenesis (Yaron et al., 2003). LH induces final gonadal maturation and ovulation more effectively by stimulating the secretion of mature steroid hormones (Yaron et al., 2003).

Therefore, artificial breeding in an indoor environment may affect the development of ovaries by blocking migration behavior. Previous studies have shown that water stimulation can significantly reduce mortality in conger eels during ovarian maturation (Meng, 2020). Studying the regulatory effect of swimming on the ovarian development of conger eel is necessary for optimizing artificial breeding techniques of conger eels. In this study, conger eels were induced to swim for 30 or 60 days through water stimulation with hormone injection and no-hormone injection. To explore the effects of flowing water stimulation on hormone regulation during the artificial reproduction of conger eel, we analyzed the relative expression levels of *FSHβ* and *LHβ* genes in the pituitary gland, as well as the changes in steroid hormones in the ovary and serum. The present study may provide a theoretical basis for the optimization of artificial reproduction technology of conger eel and other fish.

## 2 Materials and methods

### 2.1 Ethics statement

All fish samples were handled by the Animal Ethics Committee of Shanghai Ocean University (2016 NO.4) and the Regulations for the Administration of Affairs Concerning Experimental Animals approved and authorized by the State Council of the People’s Republic of China.

### 2.2 Experimental eels

The female conger eel (n = 86, 303.64 ± 10.02 g) with oocytes at the oil droplet stage and primary yolk globule stage were obtained from Shenghang Aquatic Technology Co. Ltd. (Weihai, China). The developmental stages of the female eels were determined by observing the histological sections of ovaries. Oocytes in the oil droplet stage contain numerous vacuolar small lipid droplets, while oocytes in the primary yolk globule stage exhibit light purple yolk protein granules at the cell periphery. The experiment begun in May 2021 at the company’s fish farm. The eels were acclimated in a cement tank (30.0 ± 0.5 psu, 18°C ± 0.5°C) for 2 weeks before the experiment.

A cylindrical glass fiber tank (radius = 0.75 m, height = 1.5 m, aquaculture water body = 1000 L) with a built-in pump was designed to provide circulating water flow for fish swimming (Liu et al., 2022). According to a previous study on flow velocity (0.4 ± 0.05 m/s) and the findings of the eel migration route, the swimming duration was set at 12 h per day (Hammer, 1995; Miller et al., 2011; Meng, 2020).

### 2.3 Experiment design and sampling

Eels were selected and randomly distributed into four cylindrical glass fiber tanks (n = 20, 30.0 ± 0.5 psu, 18°C ± 0.5°C), and the criteria for selecting healthy eels include appropriate size, good vitality, absence of injuries or illnesses, and a silver-white abdomen. Six acclimated eels were sampled and measured as a control group on the first day. Circulating water flow and hormone injection regime were two factors set in the following trials: A1 (no hormone injection, still water), A2 (no hormone injection, flowing water), B1 (hormone injection, still water), and B2 (hormone injection, flowing water). All experimental groups were placed in a dark environment.

A mixture of carp pituitary extract (CPE, 20 mg/kg) and human chorionic gonadotropin (HCG, 100 IU/kg) was intraperitoneally injected weekly to induce eel development of the hormone injection groups (B1 and B2), while the no hormone injection groups (A1 and A2) were injected with an equal amount of physiological saline solution until the end of the experiment. On the 30th and 60th days of the trial, six eels in each group were anesthetized using MS-222 (0.05 g/L). The weight of each eel was measured. Blood samples were collected from the bulbous arteriosus using a syringe and centrifuged at 3000 rpm for 15 min at 4°C. The Serum was frozen at −80°C. The pituitary and ovary were weighed and stored in a RNase-free tube at −80°C for Quantitative real-time PCR analysis.

### 2.4 Quantitative real-time PCR

The distinct functions of FSH and LH are determined by their respective β subunits, encoded by the *FSHβ* and *LHβ* genes. After obtaining the gene sequences of *FSHβ* (GenBank accession no. AB045157.1) and *LHβ* (GenBank accession no. AB045158.1) of conger eel on NCBI, specific primers were designed by primer software and used for quantitative real-time PCR to analyze gene expression. The *β-actin* gene served as an internal control. Before the experiment, the specificity and amplification efficiency of *β-actin* primers were verified (Table 1)**.**

**TABLE 1:** Primers’ sequences of Target Genes in qPCR from Conger eel.

Primer	Sequence of primer	Target gene
*β-actin-F*	5′- CAG​GTC​ATC​ACC​ATC​GGC​AA -3′	*β-actin*
*β-actin-R*	5′- TCC​TTC​TGC​ATT​CTG​TCG​GC -3′	
*FSHβ-F*	5′- GTT​GAT​GCT​GGC​TCC​TGC​TCT​G -3′	*FSHβ*
*FSHβ-R*	5′- ACA​CAG​GGT​CCT​GGG​TGA​AGC -3′	
*LHβ-F*	5′- GAC​AGT​CCG​TCT​GCC​AGA​TTG​C -3′	*LHβ*
*LHβ-R*	5′- GCA​CAG​GTT​ACA​GTC​ACA​GCT​CAG -3′	

Total RNA was extracted from the liver using a kit (Tiangen Biotech Co., Ltd.) according to the manufacturer’s instructions. About 1 μg of total RNA was reverse transcribed with Hifair^®^ Ⅱ first Strand cDNA Synthesis SuperMix for qPCR (gDNA digester plus) (CAT:11123 ES). Diluted cDNA (1:10) was used in all qPCR reactions. The qRT-PCR experiments were carried out in triplicate on CFX96 Touch Real-time PCR Detection System (Applied Biosystems^®^, BIO-RAD, America) using 1 μL of diluted cDNA as a template for each reaction with SYBR Green PCR Master Mix (Bio-Rad). Thermal cycling conditions included an initial heat denaturation step at 95°C for 30 s, 40 cycles at 95°C for 5 s, 60°C for 30 s, and at 95°C for 15 s. Melting curves of the PCR products were determined from 60°C to 95°C to ascertain the specificity of the amplification. Relative gene expression was calculated using the 2^−ΔΔCt^ method.

### 2.5 Ultra-high-performance liquid chromatography–tandem mass spectrometry (UPLC-MS/MS)

The extraction of steroid hormones in serum followed the method described by Dang (Dang, 2020). Specifically, 10 µL serum was homogenized with methyl tert-butyl ether (MTBE) and condensed to dry. Subsequently, 50 µL methanol (chromatographic grade) was added to dissolve the residue. After thorough shaking and mixing, the mixture was filtered through a microporous filter membrane (0.22 µm, Shanghai Amp Biotechnology Co., LTD.) into a 1.5 mL sample bottle for machine detection.

The extraction of steroid hormones from ovaries was conducted based on the protocol outlined by Ma (Ma et al., 2016). Pre-cooled methanol (chromatographic grade) was added to the ovarian tissue and thoroughly homogenized. The homogenate was then subjected to centrifugation (4°C, 10000r/min, 10min), and the supernatant was collected. Following this, the crude extract underwent solid-phase extraction using an HLB solid-phase extraction column (Shanghai Amp Biotechnology Co., LTD.). The resulting extract was evaporated nearly to dryness using a nitrogen blower at 40°C, and the volume was adjusted to 500 µL with methanol. After shaking and mixing, the extract was filtered through a microporous filter membrane (0.22 µm) into a 1.5 mL sample bottle, which was to be tested by the machine.

Calibration stock solutions of E2, cortisol (COR), 17α-hydroxyprogesterone (17α-OHP), T, and 11-Ketoltestosterone (11-KT) were prepared from Shanghai Amp Biotechnology Co., LTD. The concentration of the standard curve was 1, 5, 25, 50, 100, and 200 ng/mL.

### 2.6 Enzyme−linked immunosorbent assay (ELISA)

The E2 content in the ovarian and FSH, LH content in the serum was determined using an enzyme-linked immunosorbent assay kit (Shanghai Enzyme-linked Biotechnology Co., Ltd., China) by the instructions of the kit.

### 2.7 Statistical analysis

The UPLC-MS/MS and ELISA data were processed and imported into EXCEL for analysis using Mass Lynx V4.1 and ELISA Calc software respectively. All statistical tests were performed in SPSS 22.0 software, and graphical representations were generated using GraphPad Prism 9.0. Two-way analysis of variance (ANOVA) was used for multi-group statistical analysis. Results were expressed as Mean ± standard deviation (Mean ± SD). *p < 0.05* was considered statistically significant.

## 3 Results

### 3.1 Ovarian development

**FIGURE 1:**
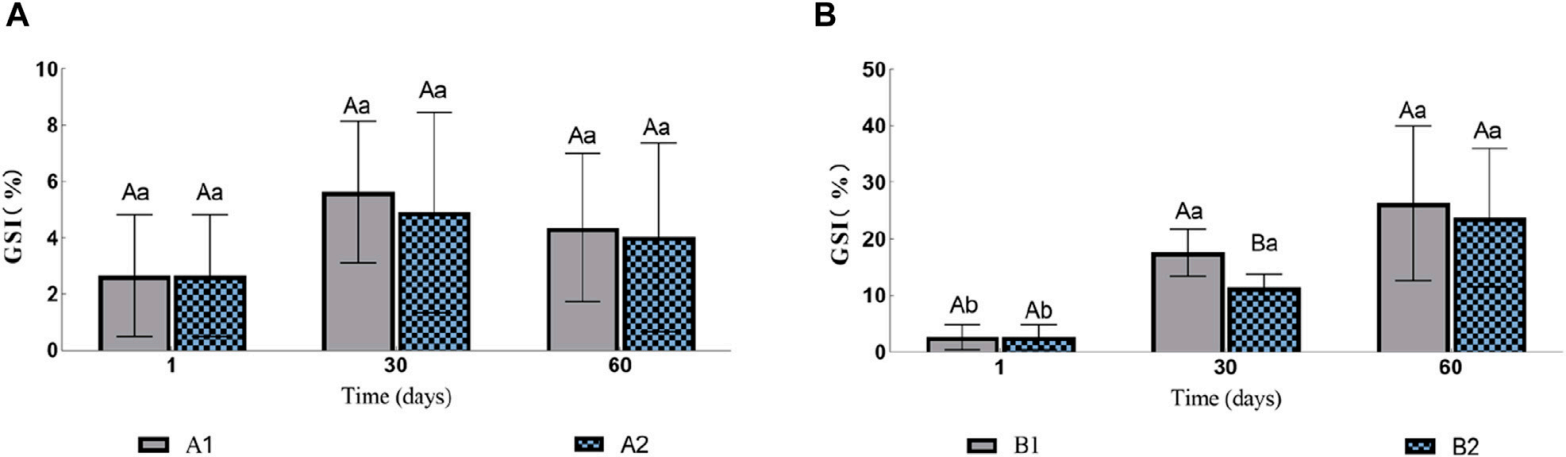
Effects of water stimulation and hormone injection on GSI of conger eel. **(A)** GSI in the no hormone group and **(B)** GSI in the hormone group. The capital letters marked in the chart indicate significant differences between the swimming group and the resting group at the same sampling time (*p < 0.05*). Lowercase letters indicate differences within the same group at different sampling periods (*p < 0.05*). A1, no hormone still water group; A2, no hormone flowing water group; B1, hormone still water group; B2, hormone flowing water group. GSI, gonadosomatic index.

In the two groups without injection (A1 and A2), there was no significant difference in gonadosomatic index (GSI, Figure 1A). The GSI of eels in groups B1 and B2 increased gradually during the experiment (Figure 1B). On the 30th day, the GSI of B1 was 17.62% ± 0.42%, which was significantly higher than that of B2 11.41% ± 4.37%. However, by the 60th day, there was no significant difference between the two groups with GSI of 26.36% ± 17.09% and 23.748% ± 12.21% respectively.

### 3.2 Changes of hormone content in ovary and serum

The concentrations of COR, 17α-OHP, T, 11-KT and E2 in serum and ovary were detected by UPLC-MS/MS, and they were well separated on XBridge C18 column. The correlation coefficients of the above standard curves were all above 0.9, indicating that there was a good linear relationship between the four hormones detected by this method and the results were reliable. The Transition one values of COR, 17α-OHP, T and 11-kT were 407.5 > 331.4, 331.4 > 96.9, 298.4 > 97.1, 303.1 > 109.1. The detection limits were 0.1, 0.04, 0.01, 0.25 ng/mL. The recoveries were 78%–98%. Therefore, this method can accurately detect five hormones of conger eel.

#### 3.2.1 COR, 17α-OHP, T, 11-KT and E2 in ovary

**FIGURE 2:**
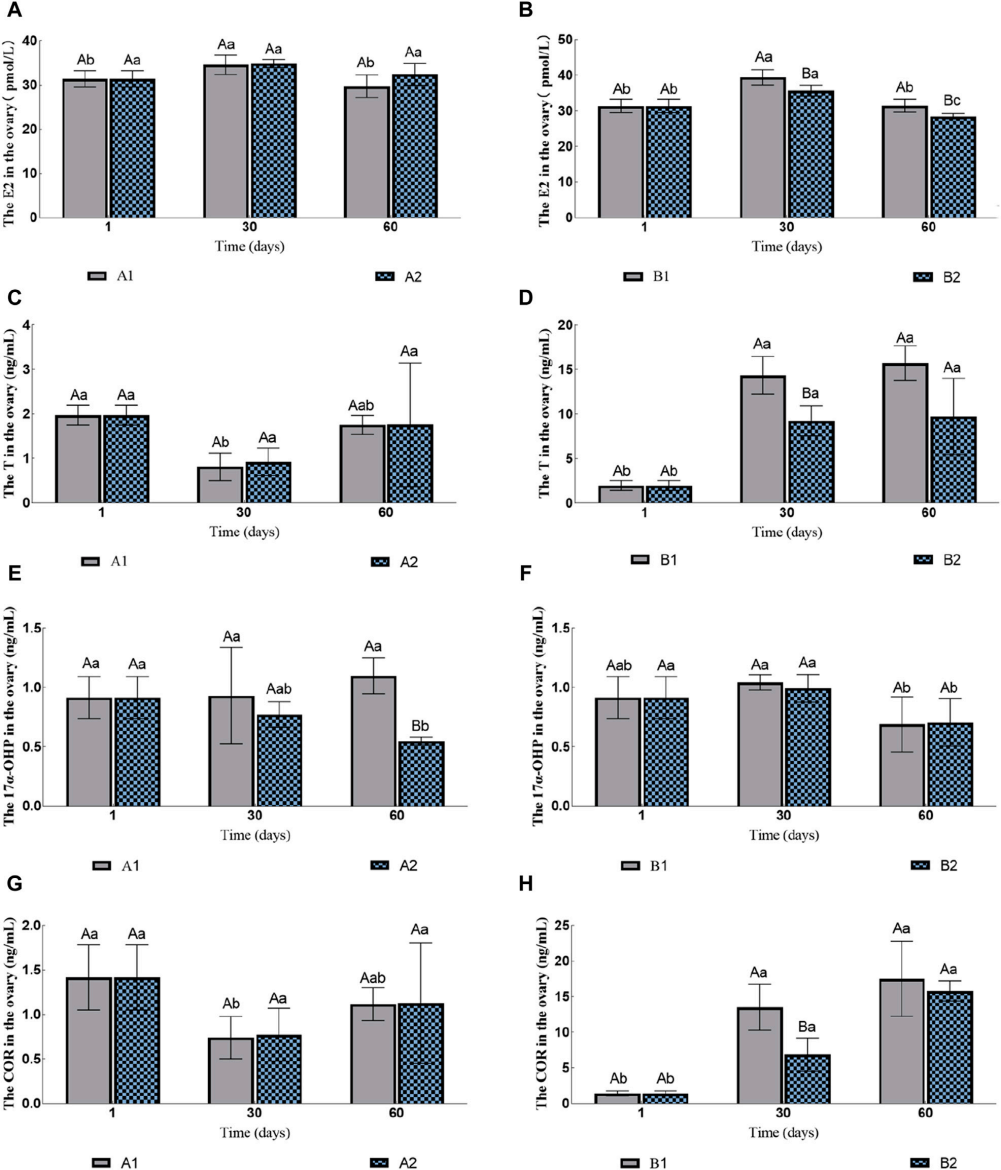
Effects of water stimulation and hormone injection on the contents of COR, 17α-OHP, T and E2 in the gonads of conger eel. **(A)** The content of E2 in ovary of no hormone group, **(B)** The content of E2 in ovary of hormone group, **(C)** The content of T in ovary of no hormone group, **(D)** The content of T in ovary of hormone group, **(E)** The content of 17α-OHP in ovary of no hormone group, **(F)** The content of 17α-OHP in ovary of hormone group, **(G)** The content of COR in ovary of no hormone group, **(H)** The content of COR in ovary of hormone group. The capital letters marked in the chart indicate significant differences between the swimming group and the resting group at the same sampling time (*p < 0.05*). Lowercase letters indicate differences within the same group at different sampling periods (*p < 0.05*). A1, no hormone still water group; A2, no hormone flowing water group; B1, hormone still water group; B2, hormone flowing water group. E2, estradiol; T, testosterone; 17α-OHP, 17α-hydroxyprogesterone; COR, cortisol.

The contents of E2 and T in the ovary of the groups A1 and A2 had no significant changes (Figures 2A, C). The content of 17α-OHP in A2 gradually decreased, while the content of 17α-OHP in A1 was significantly higher than that in A2 on the 60th day (1.10 ± 0.15 ng/mL vs. 0.55 ± 0.03 ng/mL; Figure 2E). The content of COR in A1 decreased initially and then increased, but there was no significant difference between A1 and A2 (Figure 2G). The content of 11-kT was lower than the detection limit.

The contents of E2 in both B1 and B2 increased initially and then decreased, but the group B1 was always significantly higher than B2 (Figure 2B). The contents of T and COR in B1 and B2 increased gradually; the contents of these two hormones in B1 were significantly higher than that in B2 on the 30th day (T: 14.36 ± 2.12 ng/mL vs. 9.23 ± 1.69 ng/mL; COR: 13.51 ± 3.22 ng/mL vs. 6.88 ± 2.34 ng/mL; Figures 2D,H), but no significant difference was observed on the 60th day (T: 15.71 ± 1.95 ng/mL vs. 9.75 ± 4.28 ng/mL; COR: 17.52 ± 5.25 ng/mL vs. 15.81 ± 1.42 ng/mL; Figures 2D,H). The contents of 17α-OHP decreased on the 60th day, but there was no significant difference between B1 and B2 (0.6901 ± 0.2315 ng/mL vs. 0.7047 ± 0.1995 ng/mL; Figure 2F). The contents of 11-KT in B2 were 0.97 ± 0.31 ng/mL on 30th day and there was no significant difference between the two groups on 60th day (1.10 ± 0.41 ng/mL vs. 1.05 ± 0.36 ng/mL).

#### 3.2.2 COR, 11-KT and E2 levels in serum

The contents of 17α-OHP and T in the serum of the four experimental groups were lower than the detection limit, while E2 and 11-KT had no significant change (Table 2). In all four experimental groups, COR levels were consistently significantly higher in the flowing water group than in the still water group (Table 2).

**TABLE 2:** Effects of water stimulation and hormone injection on the levels of COR, 11-KT and E2 in serum of Conger eel.

	The first day (control group)	The 30th day	The 60th day	The 30th day	The 60th day
		A1		A2	
11-KT (ng/mL)	1.31 ± 0.39^a^	1.09 ± 0.11^Aa^	1.04 ± 0.28^Aa^	1.01 ± 0.12^Aa^	1.01 ± 0.19^Aa^
E2 (ng/mL)	6.24 ± 0.69^a^	4.12 ± 1.62^Aa^	6.50 ± 2.37^Aa^	3.96 ± 1.04^Aa^	4.39 ± 1.35^Aa^
COR (ng/mL)	-	5.09 ± 2.07^B^	7.24 ± 5.88^B^	14.25 ± 6.97^A^	22.16 ± 11.36^A^
		B1		B2	
11-KT (ng/mL)	1.31 ± 0.39^a^	0.96 ± 0.18^Aa^	1.07 ± 0.38^Aa^	1.09 ± 0.32^Aa^	1.09 ± 0.25^Aa^
E2 (ng/mL)	6.24 ± 0.69^a^	7.42 ± 3.18^Aa^	4.92 ± 2.40^Aa^	5.46 ± 2.21^Aa^	5.28 ± 1.68^Aa^
COR (ng/mL)	-	7.30 ± 5.00^B^	7.24 ± 4.92^B^	22.04 ± 9.77^A^	19.11 ± 6.38^A^

Note: The capital letters marked in the chart indicate significant differences between the swimming group and the resting group at the same sampling time (*p < 0.05*). Lowercase letters indicate differences within the same group at different sampling periods (*p < 0.05*). “-" indicates that the hormone content is lower than the detection limit. A1, no hormone still water group; A2, no hormone flowing water group; B1, hormone still water group; B2, hormone flowing water group. 11-KT, 11-Ketoltestosterone; E2, estradiol; COR, cortisol.

### 3.3 *FSHβ* and *LHβ* gene expression and content changes

**FIGURE 3:**
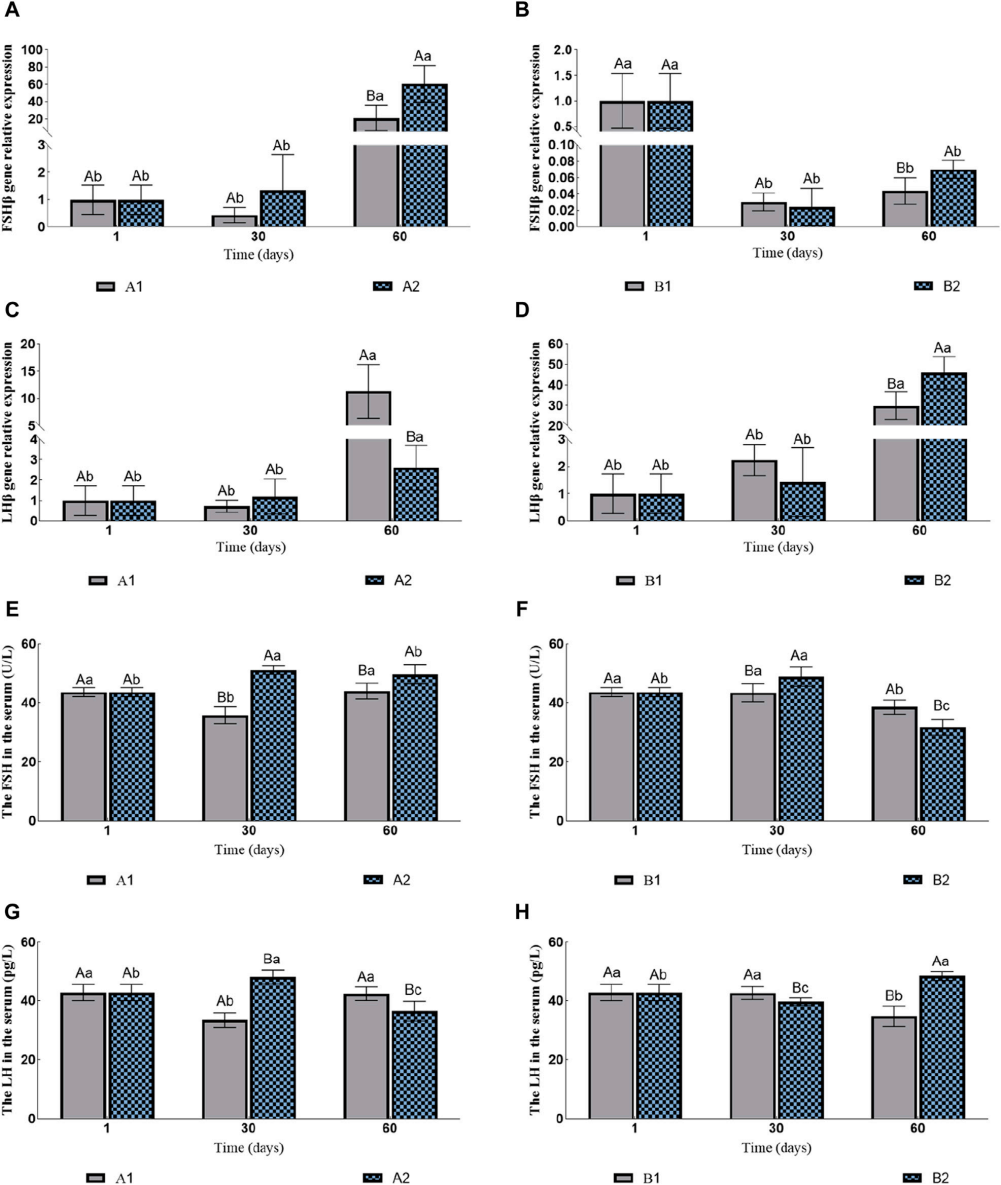
Effects of fluid stimulation and hormone injection on the expression of Follicle stimulating hormone (*FSHβ*) and luteinizing hormone (*LHβ*) genes in the pituitary, and follicle stimulating hormone (FSH) and Luteinizing hormone (LH) in the serum of conger eel. **(A)** The relative expression of *FSHβ* in the no hormone group, **(B)** The relative expression of *FSHβ* in the hormone group, **(C)** The relative expression of *LHβ* in the no hormone group, **(D)** The relative expression of *LHβ* in the hormone group, **(E)** The content of FSH in the hormone group, **(F)** The content of FSH in the hormone group, **(G)** The content of LH in the hormone group, **(H)** The content of LH in the hormone group. The capital letters marked in the chart indicate significant differences between the swimming group and the resting group at the same sampling time (*p < 0.05*). Lowercase letters indicate differences within the same group at different sampling periods (*p < 0.05*). A1, no hormone still water group; A2, no hormone flowing water group; B1, hormone still water group; B2, hormone flowing water group. *FSHβ*, Follicle-stimulating hormone beta subunit; *LHβ*, Luteinizing hormone beta subunit. FSH, Follicle-stimulating hormone; LH, Luteinizing hormone.

The expression levels of *FSHβ* in A1 and A2 increased continuously compared to those in the control group, with A2 significantly higher than A1 on the 60th day (Figure 3A). However, with hormone injection, the expression levels of *FSHβ* of B1 and B2 decreased rapidly. Similarly, B2 was significantly higher than B1 on the 60th day (Figure 3B). The expression levels of *LHβ* in all four groups increased significantly. On the 60th day, the expression levels of *LHβ* in A1 and B1 were significantly higher than A2 and B2 (Figures 3C, D), respectively.

The contents of FSH in A1 first decreased and then increased, while in A2 they first increased and then decreased. Additionally, the contents of FSH in A2 were consistently significantly higher than in A1 (the 30th day: 35.85 ± 2.81 U/L vs. 51.15 ± 1.46 U/L; the 60th day: 44.00 ± 2.61 U/L vs. 49.69 ± 3.25 U/L; Figure 3E). The contents of FSH in B1 were decreased significantly on the 60th day, while in B2 they first increased and then decreased. In addition, the contents of FSH in B2 were significantly higher than in B1 on the 30th day, and B1 was significantly higher than B2 on the 60th day (the 30th day: 43.46 ± 3.02 U/L vs. 48.91 ± 3.30 U/L; the 60th day: 38.56 ± 2.39 U/L vs. 31.72 ± 2.61 U/L; Figure 3F). The changes in the LH contents and the FSH contents in A1 and A2 were consistent. The contents of LH in A2 were significantly higher than in A1 on the 30th day, and A1 were significantly higher than A2 on the 60th day (the 30th day: 33.41 ± 2.43 pg/L vs. 47.98 ± 2.39 pg/L; the 60th day: 42.32 ± 2.31 pg/L vs. 36.39 ± 3.38 pg/L; Figure 3G). The contents of LH in B1 decreased significantly on the 60th day, while in B2 they first decreased and then increased. The contents of LH in B1 were significantly higher than in B2 on the 30th day, and B2 was significantly higher than B1 on the 60th day (the 30th day: 42.56 ± 2.18 pg/L vs. 39.62 ± 1.24 pg/L; the 60th day: 34.72 ± 3.46 pg/L vs. 48.36 ± 1.53 pg/L; Figure 3H).

## 4 Discussion

Water stimulation is crucial for some fish species, as it can stimulate ovarian development, spawning and fertilization (Li et al., 2020; Chen et al., 2021; Shu et al., 2023). This study found that water stimulation slowed down the development of ovary. Prolonged swimming can also delay ovulation in rainbow trout (*Oncorhynchus mykiss*) (Contreras, 1998). However the inhibition of vitellogenin (VTG) synthesis by water stimulation may be the main reason for the slow growth of ovary (Liu et al., 2022). Swimming can inhibit the synthesis of VTG from conger eel, a similar phenomenon also found in European eel (Palstra et al., 2010b). In the process of ovarian development, the synthesis of VTG will lead to the shedding of calcium, phosphorus, and other elements from the bone, which are then transported to the ovary. This process is extremely detrimental to long-term migration (Gao, 2011). In addition, swimming exercise suppresses oocyte development by inhibiting VTG uptake in rainbow trout, and it downregulates protein biosynthesis and energy supply functions in the ovary to conserve energy (Palstra et al., 2010a; Palstra and Planas, 2011). Therefore, water stimulation can inhibit ovarian development in migrating conger eels, conserving energy to meet their energy demands during reproduction and increasing reproductive efficiency.

*FSHβ* gene is highly expressed in the early stage of ovarian development, while *LHβ* gene is highly expressed in the late ovarian development in conger eels. The increase in hormone levels may be the cause of this phenomenon. A similar pattern was found in Japanese eel, with higher mRNA levels of *FSHβ* were found in immature fish and higher mRNA levels of *LHβ* were found in adult female and male fish treated with exogenous hormones (Yoshiura et al., 1999). In this experiment, the increase of gonad steroid content was also observed during the development of conger eel, but no change in ovarian steroid content was observed in the two groups without hormone treatment. The non-expression of *FSHβ* in the later stage of hormone injection may be due to the regulatory effect of increased steroid hormone content in the pituitary gland. Increased levels of T and E2 have been shown to significantly reduced the level of *FSHβ* mRNA in Japanese eels and European eels (Schmitz et al., 2005; Jeng et al., 2007). In the study, increased levels of hormones such as T and COR in the gonads were found in both hormone groups. These increased hormone levels may be responsible for the inhibition of FSH gene expression.

Water stimulation can promote the synthesis of FSH and LH at different stages of ovarian development in conger eels. Swimming was also observed to increase serum COR levels in this experiment. After long-term swimming, the levels of FSH and LH in conger eel significantly increased during gonadal development, which may be due to the regulation of COR on the HPG axis. When changes in the external environment put stress on the fish organisms, teleost fish adapt to the changes by promoting the synthesis of hormones such as COR through the hypothalamic-pituitary-adrenal axis (HPA) (Kr et al., 2020). COR can significantly elevate the *LHβ* mRNA content in European eels, and stimulate *FSHβ* gene expression in Atlantic cod as well (Huang et al., 1999; Krogh et al., 2019). The widespread presence of glucocorticoid receptors in both the brain and gonads of fish underscores the pivotal role of COR and other glucocorticoids in the reproductive process of fish (Teitsma, 1999; Maruska and Fernald, 2011; Ogawa and Ishwar, 2014). However, COR exerts species-specific regulatory effects on organisms, and its mechanism needs further exploration (Mommsen et al., 1999). Studies have shown that during the silver plating period of Japanese eels and European eels, the expression of *FSHβ* and *LHβ* significantly increased and effectively reduced the expression of dopamine receptors (Jeng et al., 2014). After swimming for 60 days the expression level of *FSHβ* significantly increased in fish without hormone injection, suggesting that swimming can help with the early development of fish. In zebrafish, both males and females lacking the FSH gene were able to reproduce normally, but the gonad development rate was slow, and females lacking the LH gene were unable to lay eggs (Zhang et al., 2015). When the gonads of Japanese eels mature, it is necessary to inject the hormones DHP or OHP to induce spawning in captivity (Jiang, 2012). Therefore, the observed inability of eels to ovulate naturally in our study could potentially be attributed to inadequate LH synthesis. Stimulating LH synthesis through water stimulation might prove beneficial for facilitating the final maturation and ovulation of the gonads.

Water stimulation increased the level of 11-KT in B2 ovaries on the 30th day, and reduced E2 levels in the ovaries. 11-KT is known to promote the absorption of lipid substances by oocytes (Endo et al., 2008). Similarly, swimming in European eel has also been found to increase the level of 11-KT in serum and promote the accumulation of lipid droplets in oocytes (Ginneken et al., 2007). E2 binds to estrogen receptors in the liver and promotes vitellogenin synthesis (Williams et al., 2013; Hara et al., 2016). Therefore, the decrease in E2 may lead to a decrease in vitellogenin synthesis. Palstra et al. found that swimming inhibited the expression of two VTG genes in European eel (Palstra et al., 2010b). In oviparous vertebrates, maternally derived miRNAs and hormones enter the oocytes during vitellogenesis and participate in seedling embryonic development (Groothuis and Schwabl, 2008; Tokarz et al., 2015). The contents of E2 and DHP in oocytes of European eel are negatively correlated with the quality of oocytes (Kottmann et al., 2021). Changes in serum hormone levels with no discernible pattern were observed, and were consistent with previous studies (Li, 2019). Furthermore, there are notable differences between conger eel and other eels, necessitating further study into specific regulatory mechanisms. Therefore, we should not blindly pursue the increase of hormone content related to reproduction in future reproduction work.

## 5 Conclusion

In this study, we employed qPCR to detect the relative expression levels of *FSHβ* and *LHβ* in the pituitary of the Conger eel. Additionally, we utilized ELISA and UPLC-MS/MS to assess hormone content in the serum and ovaries. FSH hormone in Conger eel only functions in the early stage of ovarian development, with water flow stimulation can significantly promote the synthesis of FSH. After hormone injection, the mRNA level of *FSHβ* gene was below detection. *LHβ* gene in Conger eel exhibits high expressions in the later stage of ovarian development, with water flow stimulation significantly promoting the synthesis of LH in the late stage of ovarian development. Water flow stimulation notably reduces the content of hormones such as COR, E2, and T in the ovaries of Conger eel while promoting COR content in the serum. This study contributes to our understanding of the impact of water flow stimulation on hormone regulation in Conger eel. Moreover, further investigation is warranted to determine whether the inhibitory effect of water stimulation on hormone content in the ovary of the Conger eel can improve the hatching rate of fertilized eggs and the survival rate of seedlings.

## Data Availability

The original contributions presented in the study are included in the article/supplementary material. The gene sequences of FSHβ and LHβ of conger eel were obtained from NCBI, and their accession numbers are AB045157.1 (https://www.ncbi.nlm.nih.gov/nuccore/AB045157.1) and AB045158.1 (https://www.ncbi.nlm.nih.gov/nuccore/AB045158.1) respectively. Further inquiries can be directed to the corresponding authors.
